# Associations between Maternal Selenium Status and Cord Serum Vitamin D Levels: A Birth Cohort Study in Wuhan, China

**DOI:** 10.3390/nu14091715

**Published:** 2022-04-20

**Authors:** Huiqing Gang, Hongling Zhang, Tongzhang Zheng, Wei Xia, Shunqing Xu, Yuanyuan Li

**Affiliations:** 1 Key Laboratory of Environment and Health, Ministry of Education & Ministry of Environmental Protection, and State Key Laboratory of Environmental Health, School of Public Health, Tongji Medical College, Huazhong University of Science and Technology, Wuhan 430030, China; ganghq342@163.com (H.G.); xiawei@hust.edu.cn (W.X.); xust@hust.edu.cn (S.X.); 2 School of Health and Nursing, Wuchang University of Technology, Wuhan 430000, China; zhanghongling@wut.edu.cn; 3 Department of Epidemiology, Brown University, Providence, RI 02912, USA; tongzhang_zheng@brown.edu

**Keywords:** cord serum 25(OH)D level, vitamin D deficiency, urinary selenium, repeated measurements

## Abstract

Serum selenium (Se) has been reported to be associated with serum 25-hydroxyvitamin D [25(OH)D], but epidemiological findings are limited in pregnant women. We aimed to assess the associations between maternal urinary Se concentrations and cord serum 25(OH)D levels. We measured urinary concentrations of Se in the first, second, and third trimesters and cord serum 25(OH)D of 1695 mother-infant pairs from a prospective cohort study in Wuhan, China. The results showed that each doubling of urinary Se concentrations in the first, second, third trimester, and whole pregnancy (average SG-adjusted concentrations across three trimesters) were associated with 8.76% (95% confidence interval (CI): 4.30%, 13.41%), 15.44% (95% CI: 9.18%, 22.06%), 11.84% (95% CI: 6.09%, 17.89%), and 21.14% (95% CI: 8.69%, 35.02%) increases in 25(OH)D levels. Newborns whose mothers with low (<10 μg/L) or medium (10.92–14.34 μg/L) tertiles of urinary Se concentrations in whole pregnancy were more likely to be vitamin D deficient (<20 ng/mL) compared with those with the highest tertile (>14.34 μg/L). Our study provides evidence that maternal Se levels were positively associated with cord serum vitamin D status.

## 1. Introduction

Selenium (Se), an essential micronutrient [1,2], is a critical component of Glutathione Peroxidase (GSH-Px), which has an irreplaceable effect on human health [3]. People usually absorb Se from the diet [4,5], which is rapidly excreted from the body mainly through urine after being metabolized [6]. Reactive oxygen species (ROS) produced by the placenta during pregnancy significantly impact placental function. As an essential trace element, Se has an irreplaceable role in antioxidants during pregnancy because of the antioxidant properties of selenoproteins [7]. The recommended Se intake is 55 μg/day [8,9], and its safe upper limit is 400 μg/day. Intake of more than 800 μg/day may lead to Se toxicity [9]: loss of hair and nails, poor neurological, skin, and dental health, garlic odor on the breath, and even paralysis [10]. Low Se status or Se deficiency has been found to be associated with adverse pregnancy outcomes such as preterm birth [11], pre-eclampsia, and hypertension during pregnancy [12]. In addition to blood (including blood, plasma, and serum), toenail, and hair [13], urinary Se is considered to be a valid biomarker for assessing Se status in humans [14]. Previous studies have shown that the time point of Se level assessment affects the accuracy of data, and pregnant women with adequate Se levels in the first trimester might be Se deficient in the third trimester. Hence, repeated measurements of urinary Se during a broader window are necessary [7,15].

Our body derives vitamin D from ultraviolet B (UV-B) radiation exposure to the skin, dietary intake, and dietary supplements [16]. Vitamin D_3,_ which was produced from the skin, and vitamin D_2_ and D_3_ from the diet are hydroxylated in the liver to produce 25-hydroxyvitamin D_2_ [25(OH)D_2_] and 25-hydroxyvitamin D_3_ [25(OH)D_3_]. Serum 25(OH)D is the key circulating metabolite of vitamin D, which has a two-week biological half-life and is a clinical measure of vitamin D status, although it will continue to undergo hydroxylation in the kidney to its active form: 1,25-hydroxyvitamin D [1,25(OH)_2_D] [17]. The impact of a newborn’s vitamin D deficiency is receiving increasing attention. Previous studies have shown that vitamin D levels in cord blood are associated with childhood diseases, such as asthma, wheezing, respiratory infections [18], and type 1 diabetes [19]. Many factors influence cord serum vitamin D levels, such as age, genetics, vitamin D intake, and outdoor activities [20]. In our previous study, a negative correlation was observed between repeatedly measured urinary cobalt (Co), vanadium (V), and thallium (Tl) concentrations during pregnancy and cord serum vitamin D status [21]. A positive correlation between serum Se concentration and vitamin D levels in humans was found in China [22]. However, the factors associated with vitamin D levels have not been thoroughly investigated. Therefore, we aimed to explore whether there is an association between Se concentrations in pregnant women and cord serum 25(OH)D levels in newborns.

Given the above, this study was based on a prospective birth cohort to assess maternal physical Se status. It used repeatedly measured Se levels to estimate the association between prenatal Se concentrations and cord serum 25(OH)D levels to provide epidemiological evidence for health care during pregnancy.

## 2. Materials and Methods

### 2.1. Study Population

Our participants were from a prospective cohort study in Wuhan, China. The pregnant women were enrolled who were: (1) <16 weeks of pregnancy with a singleton gestation at first prenatal care; (2) residents in Wuhan and willing to have prenatal visits and delivery at the target hospital; (3) willing to provide urine samples and cord blood samples and cooperate with questionnaires. From September 2013 to June 2015, a total of 3198 pregnant women entered our cohort, and 2564 provided at least one urine sample. Among them, we selected 1698 pregnant women who provided cord blood samples during delivery, excluding two pregnant women with missing prepregnancy body mass index (BMI) data and one pregnant woman with missing multivitamin D dietary supplement data, leaving 1695 pairs of mothers and infants enrolled in our study for analysis. Pregnant women in the first trimester (13.1 ± 1.1 weeks, *n* = 1579), second trimester (24.1 ± 3.4 weeks, *n* = 979), and third trimester (35.0 ± 3.1 weeks, *n* = 924) were included, and 570 participants provided urine samples in all three trimesters. Our study was approved by the Ethics Committee of Tongji Medical College of Huazhong University of Science and Technology.

### 2.2. Covariates

Covariates were obtained through interviews and medical records. Interviews were conducted using questionnaires to investigate demographic and socioeconomic characteristics, including maternal age, height, prepregnancy weight, education level, lifestyle factors (smoking, alcohol consumption, passive smoking), and vitamin D supplementation. Date of last menstrual period (LMP), gestation, pregnancy complications [e.g., gestational diabetes (GDM), pregnancy-induced hypertension (PIH), and anemia], mode of delivery, date of delivery, gestational age, and sex of the newborns were obtained from medical records. The prepregnancy body mass index (BMI) of mothers was calculated based on prepregnancy weight and height. The gestational week was calculated using the time of urine sample collection minus LMP. Weight gain during pregnancy was calculated based on prepregnancy weight versus predelivery weight. Passive smoking was defined as exposure to secondhand smoking in the home or workplace during pregnancy [23]. We divided the delivery season into Cold (December–May) and Warm (June–November) according to the climatic features of Wuhan, China: long winter and summer, short spring and autumn [21,24].

### 2.3. Urine Collection and Se Measurement

The urine samples were collected at the first, second, and third trimester of gestation in polypropylene cups and stored in sterile 5 mL pp tubes in refrigerators at −20 °C. Urinary Se, V, Co, and Tl concentrations were detected using an inductively coupled plasma mass spectrometry (ICP-MS), and the details of the method can be found in our previously published articles [25]. Urine samples were taken out of the −20 °C refrigerators and brought to room temperature before determination. After being mixed thoroughly with a turbine, urine samples were added to 1.2% (*v*/*v*) nitric acid overnight, sonicated at 40 °C for 1 h before formal detection on the machine, and finally analyzed in helium mode. Human urine specimens (SRM2670a) were used as external quality controls to evaluate the accuracy of ICP-MS in each batch of experiments. A blank sample (1.2% (*v*/*v*) HNO_3_) was added after each batch of urine samples was analyzed, and the line was flushed to control potential contamination. The limit of detections (LODs) for urinary Se, V, Co, and Tl were 0.224, 0.002, 0.010, and 0.020 µg/L, respectively, and the intraday variability of Se, V, Co, and Tl detected in urine samples was 0.553%−1.118%. Se level for one urine sample was below the LOD of Se. The interday variability was 0.272%−0.816%. Concentrations of Se, V, Co, and Tl were adjusted for variation in dilution by urinary specific gravity (SG) through the following equation: P_c_ = P_i_ [(SG_m_ − 1)/(SG_i_ − 1)], P_c_ is the SG-adjusted urinary Se, V, Co, and Tl concentration (ng/mL), P_i_ is the observed urinary Se, V, Co, and Tl concentrations (μg/L), SG_m_ is the median SG for the urine samples of each trimester, and SG is the specific gravity of each urine sample. SG was by a pocket refractometer while preparing urinary samples for analysis (Atago PAL-10S;Atago, Tokyo, Japan). In our study, 11 pregnant women had missing urinary specific gravity data in the first trimester, 10 in the second trimester, and six in the third trimester.

### 2.4. Cord Serum Collection and 25(OH)D Analyses

Details on the processing of cord blood samples and the detection of cord serum vitamin D were described in our previous article [21]. Cord blood samples are obtained immediately after delivery, centrifuged to extract cord serum, and stored in a −80 °C refrigerator prior to the determination. Liquid chromatography and triple quadrupole mass spectrometry couples (LC-MS/MS) were used to determine the levels of 25(OH)D_2_ and 25(OH)D_3_ in cord serum. The intrabatch and interbatch coefficients of variation were less than 15%. The detection limits (LODs) for 25(OH)D_2_ and 25(OH)D_3_ were 0.5 and 1.0 ng/mL, respectively. The concentrations of 25(OH)D were equated to the sum of 25(OH)D_2_ and 25(OH)D_3_.

### 2.5. Statistical Analysis

Urinary Se was replaced as LOD/2 if the concentration was below the value of LOD. Median, upper quartile, lower quartile, 25th percentile, and 75th percentile were used to describe the distributions of urinary Se concentrations at the first, second, and third trimester of gestation and whole pregnancy (averaged SG-adjusted concentrations across three trimesters) were also analyzed. The concentrations of urinary Se and cord serum 25(OH)D were naturally ln-transformed because the distributions were right-skewed. Intraclass correlation coefficient (ICC) was calculated using a linear mixed model to examine the reproducibility of participants’ SG-adjusted urinary Se levels in the first, second, and third trimesters.

We explored the association between the whole pregnancy (averaged SG-adjusted concentrations across three trimesters) urinary Se levels of pregnant women and cord serum 25(OH)D by fitting a generalized linear model (GLM). Furthermore, generalized estimating equations models (GEE) were used to investigate associations between repeatedly measured urinary Se levels and cord serum 25(OH)D. We also assessed the association between categorical variables based on tertile distribution of average SG-adjusted urinary Se levels in all three trimesters with newborn’s vitamin D deficiency using a GLM model (cord serum total 25(OH)D concentration < 20 ng/mL was considered vitamin D deficiency) [26]. We calculated the percent change of cord serum vitamin D for per doubling maternal urinary Se concentrations increase using the formula: percent change (%Δ) = [e^(ln2×β)^ − 1], where β was the coefficient from GLM and GEE models [27].

Confounders were introduced based on biological and statistical considerations. Bivariate summary analyses (*p* < 0.1) were applied to all variables. Urinary metals (V, Co, TL) concentrations were added to the model as covariates. Only one pregnant woman smoked during pregnancy, and no one reported drinking, so smoking and drinking during pregnancy were not added to the model as covariates. The final covariates included in the model were: maternal age, prepregnancy BMI, season of birth, mode of delivery, gestational weight gain, passive smoking before or during pregnancy, and multivitamin supplement use during pregnancy.

Season of birth has been considered to be associated with cord serum 25(OH)D levels in previous studies [21,28]. Therefore, our analyses were stratified by infant birth season (warm or cold) and performed separately.

Many previous studies have linked cord serum 25(OH)D levels to pregnancy complications such as gestational diabetes mellitus (GDM), pregnancy-induced hypertension (PIH), and anemia [20]. Therefore, a sensitivity analysis was carried out among pregnant women without these diseases to validate the robustness of our results.

This study performed data analyses with version 9.4 Statistical Analysis System (SAS; SAS Institute Inc., Cary, NC, USA; version 9.4). All tests were bilateral, and *p* values < 0.05 were defined as statistically significant.

## 3. Results

### 3.1. Characteristics of the Study Population

Table 1 lists the main characteristics of our participants. The average age of the 1695 pregnant women in this study was 28.37. Most of the pregnant women were primigravida (86.49%). About half of the women had an education level above high school (48.26%). Mothers who took multivitamin supplements during pregnancy were almost ten times more than those who did not take them. Thirteen pregnant women drank alcohol before pregnancy, and none of them continued during pregnancy. Thirteen pregnant women smoked before pregnancy, one woman continued to smoke during pregnancy, and 32.04% smoked passively before six months of pregnancy or during pregnancy. As for health status during pregnancy, 35 mothers had pregnancy-induced hypertension (PIH), 102 pregnant women had gestational diabetes mellitus (GDM), and 68 pregnant women had anemia. Of the newborns, 55.69% were born in the warm season, 53.81% were male, and 53.22% were delivered by cesarean section.

### 3.2. Distributions and Variability of Maternal Urinary Se and Cord Serum 25(OH)D Concentrations

The urinary Se concentrations of pregnant women are shown in Table 2. The concentrations (median (5th percentile, 95th percentile)) of urinary Se adjusted by SG in pregnant women was 17.82 (8.48, 51.58) μg/L for the first trimester, 10.39 (5.08, 26.18) μg/L for the second trimester, 11.51 (5.33, 35.18) μg/L for the third trimester, and 12.63 (7.44, 22.54) μg/L for whole pregnancy (average concentrations across three trimesters). The median value (5th percentile, 95th percentile) of 25(OH)D concentrations was 21.10 (5.53, 49.98) ng/mL. The concentration distribution of other urinary metals (V, Co, Tl) can be seen in Supplementary Material Table S2. The intraclass correlation coefficients (ICC) (Supplementary Material Table S1) of SG-adjusted urinary Se, V, Co, and Tl were 0.48 (95% CI: 0.44, 0.52), 0.40 (95% CI: 0.36, 0.45), 0.24 (95% CI: 0.20, 0.29), and 0.41 (95% CI: 0.37, 0.45), ranged from 0.24 to 0.48, which indicated poor to fair reproducibility.

### 3.3. Individual Urinary Se and Cord Serum 25(OH)D

Table 3 shows the association between repeated measurements of urinary Se and cord serum 25(OH)D concentrations. In model 1, we found a positive association between urinary Se and cord serum 25(OH)D concentrations in the second trimester, with per doubling of urinary Se concentration increasing cord serum 25(OH)D concentrations by 8.95% (95% confidence interval (CI): 3.20%, 15.04%). After concentrations of V, Co, and Tl during each trimester were included in model 2 simultaneously, urinary Se concentrations were associated with cord serum 25(OH)D concentrations in the first trimester, second trimester, and the third trimester with each twofold increase in urinary Se being associated with 8.76% (95% CI: 4.30%, 13.41%), 15.44% (95% CI: 9.18%, 22.06%), and 11.84% (95% CI: 6.09%, 17.89%) increase in cord serum 25(OH)D. A generalized linear model (GLM) was used to analyze the relationship between urinary Se concentrations in whole pregnancy and cord serum 25(OH)D concentrations. In model 1 and model 2, per doubling of average urinary Se levels increase were associated with 16.86% (95% CI: 5.33%, 29.67%), 21.14% (95% CI: 8.69%, 35.02%) increase in cord serum 25(OH)D.

### 3.4. Association between Urinary Se Levels and Newborns’ Vitamin D Deficiency

As shown in Table 4 in the analysis of prenatal Se levels and newborns’ vitamin D deficiency, among all mother-infant pairs, vitamin D deficiency risk increased 59% in the low [0.59 (95% CI: 0.12, 1.06)] and 52% in the medium [0.52 (95%CI: 0.06, 0.97)] tertiles of urinary Se concentrations vs. the high group (*p* for trend = 0.01). After stratified by the season of birth, a significant association between urinary Se levels and vitamin D deficiency was observed among newborns who were born in the cold season [0.91 (95% CI: 0.19, 1.64) in the low group and 1.02 (95% CI: 0.28, 1.77) in medium group vs. high group, *p* for trend = 0.02], but not in the warm season (*p* > 0.05).

### 3.5. Sensitive Analysis

After excluding pregnant women with GDM, PIH, or anemia, the results were almost unchanged (Table S3), indicating that the association between urinary Se concentrations and cord serum 25(OH)D would not be influenced by these diseases.

## 4. Discussion

Based on a prospective cohort study design, we included 1695 pregnant women in Wuhan to investigate the specific trimester Se levels in urine during pregnancy and explore the association between urinary Se level and cord serum 25(OH)D status. For our participants, low levels of mothers’ urinary Se were observed to be a risk factor for newborns’ vitamin D deficiency (<20 ng/mL), and the negative effect seems to be more prominent in newborns who were born during the cold season.

The urinary Se concentration adjusted by urinary specific gravity (median) of the participants in our study (Wuhan, Central China) was 17.82 μg/L for the first trimester (*n* = 1528), 10.9 μg/L for the second trimester (*n* = 969), 11.51 μg/L for the third trimester (*n* = 918), and 12.63 μg/L for whole pregnancy (average SG-adjusted concentrations across three trimesters) (*n* = 570). The median value of unadjusted urinary Se concentration in the general population of northeastern China was 17 μg/L, while our data for pregnant women were lower (median = 10.36–15.96 μg/L) [29]. The data we obtained were lower than in Mexico (third trimester: 35.8 μg/L, *n* = 132), Greece (second trimester: 22.8 μg/L (unadjusted), *n* = 176 [30]; 22 μg/L, *n* = 575 [31]), but higher than in Bangladesh (first trimester: 9.0 μg/L, *n* = 74; 6.7, *n* = 152) [32].

Only one paper from China showed a positive association between serum Se and vitamin D in menopausal women with osteoporosis [22]. As far as we know, our study is the first epidemiological study to investigate the association of maternal urinary Se concentrations with cord serum 25(OH)D levels. Our previous study suggested the potential effects of prenatal exposure to metals (V, Co, and Tl) on decreased cord serum 25(OH)D concentrations [21]. After controlling for the above three urinary metals concentrations, the effect of urinary Se on cord serum 25(OH)D was increased, indicating a potential additive effect of prenatal exposure to V, Co, and Tl, and Se status during pregnancy, so it is required to adjust the metal concentration. Our finding suggests robust relationships between pregnant Se levels and cord serum 25(OH)D status, which are unlikely to be false positives.

We also performed a sensitivity analysis, and the association remained significant after excluding pregnant women diagnosed with GDM, PIH, and anemia during pregnancy. The results were similar to the main analyses, indicating the results were independent of these diseases.

When we stratified the analysis according to the delivery season, we observed that a low level of urinary Se status during the cold season was associated with a newborn’s vitamin D deficiency. The potential mechanism is unknown, but a possible reason is that mothers who give birth during the warm season have more exposure to the sun’s ultraviolet (UV) rays. The 7-dehydrocholesterol in the skin is converted to previtamin D_3_ upon penetration by solar UV-B radiation (wavelength: 290–315 nm) and then rapidly converted to vitamin D_3_ [16]. Consequently, pregnant women who give birth in the warm season (Jun–Nov) are less likely to be vitamin D deficient than in the cold season (Dec–May), and the excessive vitamin D produced in the body is broken down by UV-B exposure. Therefore, Se is more protective of vitamin D in mothers who give birth in the cold season.

In our study, we found a positive association between Se levels during pregnancy and cord serum 25(OH)D levels. We assumed the potential mechanism for this effect might be the protective effect of Se on the liver, which is known to be a protective factor against liver necrosis [33]. The liver is the site of vitamin D hydroxylation [17]. Se is considered to have an excellent antioxidant capacity and protects intracellular structures from oxidative damage. Selenoprotein is also involved in synthesizing mitochondria, stabilizing the endoplasmic reticulum, and plays a role in preventing oxidative stress in the placenta [2,34].

Our study has some advantages. The first strength relies on its prospective cohort design and the repeated measurements of urinary Se concentrations in the first, second, and third trimesters during pregnancy, which allowed us to explore the trimester-specific effects of Se levels and cord serum 25(OH)D and identify the critical windows of Se supplement. Additionally, to the best of our knowledge, it is the first time to explore the association between urinary Se levels and cord serum 25(OH)D. Then we had a large sample size for the analysis; we had 1695 pregnant women added to the research.

Nevertheless, some limitations should be acknowledged. First, we measured Se levels in urinary samples; however, blood Se is considered the best biomarker for measuring Se status in humans [13,35]. However, it has also been suggested that urinary Se is a valid biomarker for assessing Se status in humans [14]. Secondly, only an epidemiological association was observed, and we were unable to provide a mechanistic aspect to the study. Third, vitamin D intake in the daily diet of pregnant mothers was missing in our study, but previous studies have suggested that there is no association between dietary vitamin D intake and cord blood vitamin D levels, possibly due to low dietary intake of vitamin D [28,36]. In addition, the previous studies found that 25(OH)D concentrations increased from the first trimester to the third trimester [37]. However, in our study, urinary selenium levels were lowest in mid-pregnancy and highest in early pregnancy. In our study, we only focused on cord serum vitamin D levels and did not test serum vitamin D in early, mid, and late pregnancy, so this point is also a drawback of our study. The prevalence of GDM in the entire population of our cohort was approximately 9.8%, which can be found in our previously published article [25]. However, our outcome indicator in this study was cord serum vitamin D. Therefore, our study population was pregnant women who provided at least one urine as well as cord blood sample during pregnancy, and we hypothesized that people in this population are more focused on lifestyle and health care during pregnancy. Conditions such as GDM are widely noted pregnancy complications, which may explain the low prevalence of these three pregnancy complications in our population. Future research is warranted to explore the underlying mechanisms.

## 5. Conclusions

In summary, our study shows that urinary Se concentration during pregnancy is a protective factor on cord serum 25(OH)D level. A low level of urinary Se is a risk factor for vitamin D deficiency in newborns.

## Figures and Tables

**Table 1:** Characteristics of mother-infant pairs (*n* = 1695).

Characteristics	*n*	Mean ± SD or Percent
Age (years)		28.37 ± 3.29
≤24	150	8.85
25–29	1036	61.12
30–34	420	24.78
≥35	89	5.25
Prepregnancy BMI (kg/m^2^)		20.76 ± 2.75
Underweight (<18.5)	336	19.82
Normal (18.5–23.9)	1151	67.91
Overweight (≥24)	208	12.27
Gestational weight gain (kg)		16.39 ± 4.78
Parity		
Multiparous	229	13.51
Nulliparous	1466	86.49
Educational level		
High school and below	877	51.74
More than high school	818	48.26
Multivitamin supplement use during pregnancy		
No	153	9.03
Yes	1542	90.97
Passive smoking before/during pregnancy		
No	1152	67.96
Yes	543	32.04
Drinking before pregnancy		
No	1682	99.23
Yes	13	0.77
Gestational age (week)		39.30 ± 1.20
Mode of delivery		
Vaginal delivery	793	46.78
Cesarean delivery	902	53.22
Season of birth		
Cold (December–May)	751	44.31
Warm (June–November)	944	55.69
Infant sex		
Male	912	53.81
Female	783	46.19
PIH		
No	1660	97.94
Yes	35	2.06
GDM		
No	1593	93.98
Yes	102	6.02
Anemia		
No	1627	95.99
Yes	68	4.01

**Table 2:** The distributions of maternal urinary Se and cord serum 25(OH)D concentrations during pregnancy.

Concentrations	*n*	Percentiles				
		5th	25th	50th	75th	95th
Urinary Se (μg/L)						
Unadjusted						
first trimester	1539	3.16	8.99	15.96	29.17	64.07
second trimester	979	2.72	6.13	10.36	17.54	36.47
third trimester	924	3.00	6.37	10.63	18.66	42.65
Whole pregnancy *	570	4.79	8.30	12.30	17.81	27.36
SG-adjusted						
first trimester	1528	8.48	13.27	17.82	26.52	51.58
second trimester	969	5.08	7.79	10.39	14.14	26.18
third trimester	918	5.33	8.50	11.51	16.01	35.18
Whole pregnancy *	570	7.44	10.09	12.63	15.38	22.54
Cord serum 25(OH)D (ng/mL)						
25(OH)D_2_	1695	0.25	0.96	1.15	1.47	2.51
25(OH)D_3_	1695	4.29	11.27	19.79	31.09	48.53
Total 25(OH)D	1695	5.53	12.39	21.10	32.47	49.98

* Average concentrations across three trimesters.

**Table 3:** Associations of maternal urinary Se concentrations and cord serum 25(OH)D level.

Variable	Model 1	*p*-Value	Model 2	*p*-Value
	%Δ (95%CI)		%Δ (95%CI)	
Selenium				
1st trimester ^a^	0.79 (−2.94, 4.67)	0.683	8.76 (4.30, 13.41)	<0.0001
2nd trimester ^a^	8.95 (3.20, 15.04)	0.002	15.44 (9.18, 22.06)	<0.0001
3rd trimester ^a^	3.18 (−1.82, 8.42)	0.217	11.84 (6.09, 17.89)	<0.0001
Whole pregnancy ^b^	16.86 (5.33, 29.67)	0.003	21.14 (8.69, 35.02)	0.0005

CI, confidence interval. ^a^ Generalized estimating equation model, model 1 adjusted for maternal age, prepregnancy BMI, season of birth, mode of delivery, gestational weight gain, passive smoking before/during pregnancy, and multivitamin supplement use during pregnancy. Model 2 adjusted for covariates in model 1 and SG-adjusted metals levels (Vanadium, Cobalt, Thallium). ^b^ Generalized linear model, model 2 adjusted for covariates in model 1 and additionally adjusted average SG-adjusted concentrations of each metal (Vanadium, Cobalt, Thallium) across different trimesters.

**Table 4:** Association between urinary Se levels and newborns’ vitamin D deficiency.

Se Concentrations (μg/L SG)	All ^a^	*p*-Value	Cold Season ^b^	*p*-Value	Warm Season ^c^	*p*-Value
	β (95%CI)		β (95%CI)		β (95%CI)	
Low (<10.92)	0.59 (0.12, 1.06)	0.014	0.91 (0.19, 1.64)	0.013	0.52 (−0.12, 1.17)	0.111
Medium (10.92–14.34)	0.52 (0.06, 0.97)	0.026	1.02 (0.28, 1.77)	0.007	0.30 (−0.31, 0.91)	0.334
High (>14.34)	Reference		Reference		Reference	
p for trend		0.015		0.018		0.109

^a^ Generalized linear model, adjusted for maternal age, prepregnancy BMI, season of birth, mode of delivery, gestational weight gain, multivitamin supplements use during pregnancy, passive smoking before/during pregnancy, and SG-adjusted metals levels (Vanadium, Cobalt, Thallium). ^b^ Infants born in the cold season (Dec–May), adjusted for covariates except season of birth in a. ^c^ Infants born in the warm season (June–November), adjusted for covariates except season of birth in a.

## Data Availability

The data presented in this study are available upon request from the corresponding author.

## Supplementary Materials

The following supporting information can be downloaded at: https://www.mdpi.com/article/10.3390/nu14091715/s1, Table S1: Intraclass correlation coefficients (ICC) of SG-adjusted urinary metals concentrations in different trimesters, Table S2. The distributions of metal concentrations during pregnancy, Table S3. Sensitive analysis.
