# Supplementary material for: Associations between Maternal Selenium Status and Cord Serum Vitamin D Levels: A Birth Cohort Study in Wuhan, China
Source: Nutrients. 2022 Apr 20;14(9):1715. doi: 10.3390/nu14091715 (PMC9104068; doi:10.3390/nu14091715)
Supplement: Supplementary file 1 [file nutrients-14-01715-s001.zip › nutrients-1662537-supplementary.pdf]

Supplementary Material

Associations between Maternal Selenium Status and Cord Serum Vitamin D Levels: A Birth Cohort Study in Wuhan, China

Huiqing Gang [1], Hongling Zhang [2], Tongzhang Zheng [3], Wei Xia [1], Shunqing Xu [1], Yuanyuan Li [1,*]

Table of Contents

Table S1 Intraclass correlation coefficients (ICC) of SG-adjusted urinary Se, V, Co, and Tl concentrations in different trimesters.

Table S2. The distributions of metals (V, Co, Tl) concentrations during pregnancy.

Table S3. Sensitive analysis.

Table S1 Intraclass correlation coefficients (ICC) of SG-adjusted urinary Se, V, Co, and Tl concentrations in different trimesters.

SG-adjusted urinary metals	ICC	95%CI
Selenium	0.48	(0.44,0.52)
Vanadium	0.40	(0.36,0.45)
Cobalt	0.24	(0.20,0.29)
Thallium	0.41	(0.37,0.45)

Table S2. The distributions of metals (V, Co, Tl) concentrations during pregnancy.

		Percentiles				
**Urinary Metals(μgg/L)**	***n***	**5th**	**25th**	**50th**	**75th**	**95th**
**V**						
**first trimester**	1539	0.26	0.67	1.05	1.47	2.45
**second trimester**	979	0.28	0.55	0.88	1.23	1.83
**third trimester**	924	0.28	0.56	0.86	1.20	2.20
**Whole pregnancy** [a]	570	0.36	0.72	0.93	1.17	1.74
**Co**						
**first trimester**	1539	0.07	0.18	0.29	0.46	1.02
**second trimester**	979	0.11	0.24	0.41	0.71	1.75
**third trimester**	924	0.16	0.36	0.65	1.18	2.72
**Whole pregnancy** [a]	570	0.20	0.32	0.45	0.69	1.15
**Tl**						
**first trimester**	1539	0.08	0.23	0.38	0.60	1.20
**second trimester**	979	0.08	0.19	0.32	0.48	0.88
**thirs trimester**	924	0.07	0.17	0.28	0.44	0.93
**Whole pregnancy** [a]	570	0.14	0.24	0.33	0.45	0.64

V—Vanadium; Co—Cobalt; Tl—Thallium. [a] Average SG-adjusted concentrations across different trimesters.

Table S3. Sensitive analysis. [*]

Exposure	Model 1	*p*-Value	Model 2	*p*-Value
	%Δ (95%CI)		%Δ (95%CI)	
first trimester [a]	0.84 (-3.14, 4.98)	0.6839	9.18 (4.37, 14.20)	0.0001
second trimester [a]	8.31 (2.07, 14.93)	0.0084	14.54 (7.80, 21.72)	<0.0001
third trimester [a]	3.53 (-1.81, 9.17)	0.1996	12.40 (6.24, 18.92)	<0.0001
Whole pregnancy [b]	14.38 (2.12, 28.11)	0.0203	19.24 (6.02, 34.11)	0.0033

CI—confidence interval. [*] The mothers with GDM, PIH, and anemia among our participants were excluded. [a] Generalized estimating equation model, model 1 adjusted for maternal age, prepregnancy BMI, the season of birth, mode of delivery, gestational weight gain, passive smoking before/during pregnancy, and multivitamin supplement use during pregnancy. Model 2 adjusted for covariates in model 1 and SG-adjusted metals levels (Vanadium, Cobalt, Thallium). [b] Generalized linear model, model 2 adjusted for covariates in model 1 and SG-adjusted average concentrations of each metal (V, Co, Tl) across different trimesters.
